# Retinal Ischemic Perivascular Lesions: An Exploratory Study of Their Potential as Biomarkers for Cardiovascular Disease

**DOI:** 10.3390/jcm14113837

**Published:** 2025-05-29

**Authors:** Manuel Moriche Carretero, Ana de los Reyes Sánchez Parejo, Marc Biarnés Pérez, Remedios Revilla Amores, Ángel Pérez Gómez, Clara Martinez-Perez

**Affiliations:** 1 Ophthalmology Department, Infanta Sofía University Hospital, 28703 Madrid, Spain; mmoriche@gmail.com (M.M.C.); remerevilla@gmail.com (R.R.A.); 2 Facultad de Ciencias de la Salud, Universidad Europea de Madrid, 28670 Madrid, Spain; apg1907@gmail.com; 3 OMIQ Research, 08029 Barcelona, Spain; marcmbp@gmail.com; 4 Instituto Superior de Educação e Ciências de Lisboa (ISEC Lisboa), Alameda das Linhas de Torres, 179, 1750-142 Lisboa, Portugal; clara.perez@iseclisboa.pt

**Keywords:** RIPLs, heart disease, retina, optical coherence tomography, ischemic

## Abstract

**Background/Objectives**: This exploratory study aimed to assess the prevalence of retinal ischemic perivascular lesions (RIPLs) in individuals with cardiovascular disease (CVD) or associated risk factors and to investigate their potential role as non-invasive biomarkers of systemic ischemia using optical coherence tomography (OCT). **Methods**: A prospective observational study was conducted between July and October 2022. A total of 665 participants aged 40–90 years underwent macular OCT imaging using the Topcon Maestro 2 system. Participants were classified into two groups: those with ischemic CVD or risk factors (*n* = 297) and healthy individuals without cardiovascular conditions (*n* = 368). RIPLs were defined by inner nuclear layer thinning and outer nuclear layer expansion in perivascular regions and were identified by masked consensus of three independent evaluators. **Results**: The overall prevalence of RIPLs was 0.75% (five cases), exclusively observed in the diseased group (1.68%), with no cases identified among healthy individuals (*p* = 0.044). Stratified analysis showed an increase in RIPL prevalence with age, reaching 2.24% in the 70–79 years cohort. Statistically significant associations were found between RIPLs and hypertension, dyslipidemia, ischemic heart disease, and thrombosis (all *p* < 0.001). No significant association was observed with sex, myocardial infarction, or RIPL presence as an independent predictor (*p* = 0.08). **Conclusions**: Their identification through OCT during routine ophthalmologic examinations highlights a possible new avenue for early cardiovascular risk stratification. Nevertheless, the extremely low number of RIPL cases detected (only five out of six hundred and sixty-five participants; 0.75%) significantly limits the statistical power of the analysis and precludes strong conclusions. These findings should be regarded as preliminary and hypothesis-generating, requiring confirmation in larger, more diverse populations.

## 1. Introduction

Cardiovascular disease (CVD) remains the leading cause of mortality and disability worldwide, accounting for approximately 125,000 deaths and five million hospital admissions annually in Spain alone [1,2]. Globally, the World Health Organization reports that among the 17 million premature deaths occurring in individuals under 70 years of age due to non-communicable diseases in 2019, 38% were attributed to CVD [3]. Despite the known benefits of lifestyle modifications and appropriate medical interventions [3], early detection of subclinical cardiovascular dysfunction remains a major clinical challenge. Indeed, many individuals experience myocardial infarction or stroke as their first manifestation of disease, often without prior diagnosis [4,5,6].

In this context, the retina offers a unique, non-invasive window into systemic microvascular health. Due to its direct vascularization, lack of collateral circulation, and high metabolic demand, the retina is exquisitely sensitive to even subtle changes in blood flow [7,8]. It comprises two main vascular layers: the superficial capillary plexus, supplying the inner retina, and the deep capillary plexus, supplying the inner nuclear layer [7,8]. The outer retina, being avascular, relies on diffusion from the choriocapillaris, further heightening its vulnerability to ischemic injury. Systemic ischemic conditions, such as atherosclerosis, carotid artery stenosis, and heart failure, have been associated with retinal microvascular abnormalities [9,10,11], highlighting the retina’s role as a sentinel of systemic vascular compromise.

Given its unique vascular architecture and accessibility through non-invasive imaging, the retina has emerged as a valuable structure for studying systemic vascular disease [12]. Optical coherence tomography (OCT), in particular, allows high-resolution visualization of inner retinal layers and has become increasingly used not only in ophthalmology but also in systemic disease screening [13]. Numerous studies have linked retinal microvascular abnormalities—such as changes in vessel caliber, microaneurysms, or cotton wool spots—to hypertension, diabetes, stroke, and chronic kidney disease. These findings have prompted interest in establishing the retina as a surrogate marker for microvascular health across different organ systems. The identification of subtle ischemic patterns, such as retinal ischemic perivascular lesions (RIPLs), further expands this concept by offering potential insight into chronic or resolved systemic ischemic processes, even before overt clinical events arise [14]. As such, RIPLs may represent a subclinical vascular footprint accessible through routine OCT imaging.

When ischemic injury occurs, the pattern of retinal involvement depends on the severity and localization of vascular compromise. Severe occlusions primarily affect the inner retinal layers, while milder ischemic events selectively impact the middle retina. This selective middle retinal ischemia manifests as paracentral acute middle maculopathy (PAMM), characterized by hyperreflective bands at the level of the inner nuclear layer on optical coherence tomography [11,12,13,14,15]. Chronic sequelae of retinal ischemia have recently been recognized as retinal ischemic perivascular lesions (RIPLs), first formally described in 2021 [14]. RIPLs are defined by focal thinning and atrophy of the inner nuclear layer adjacent to retinal vessels, often accompanied by an upward expansion of the outer nuclear layer [14,16,17,18,19,20]. While PAMM represents an acute ischemic insult, RIPLs reflect chronic or resolved ischemic injury.

Importantly, the structural changes detected by OCT can precede functional impairment, making this technology particularly useful in early disease identification. In systemic conditions where subclinical microvascular compromise may exist for years before clinical presentation—such as in atherosclerosis or heart failure—RIPLs could reflect cumulative ischemic damage. Furthermore, unlike other retinal findings that may be transient or confounded by acute systemic states, RIPLs appear to represent stable, chronic lesions that persist over time, providing a potential historical record of vascular stress. This stability may offer diagnostic advantages in population screening, especially when evaluating asymptomatic individuals or stratifying cardiovascular risk in primary care settings. As research on retinal biomarkers advances, their incorporation into multidisciplinary approaches for systemic disease monitoring may become increasingly relevant [14].

Beyond their ocular implications, RIPLs have attracted growing interest as potential biomarkers of systemic vascular pathology. Several studies have demonstrated that RIPLs occur with greater frequency in individuals with coronary artery disease, myocardial infarction, atrial fibrillation, and carotid artery stenosis compared to healthy controls [15,19,21,22]. Notably, patients with cardiovascular conditions exhibit an increased number of RIPLs per eye, averaging approximately 2–3 lesions, compared to near absence in healthy populations [15,19]. These findings suggest that RIPLs may serve as indicators of underlying systemic microvascular disease, accessible through non-invasive OCT imaging, and may assist in cardiovascular risk stratification.

However, despite these promising associations, most studies to date have focused on highly selected high-risk cohorts, limiting the generalizability of findings. Data regarding the prevalence of RIPLs in broader, unselected general populations remain scarce. A clearer understanding of their epidemiological distribution could enhance the potential role of RIPLs as screening biomarkers for systemic vascular disease in routine clinical practice.

Accordingly, this study was designed as an exploratory observational analysis aimed at systematically assessing the prevalence and distribution of RIPLs in a general adult screening population and exploring their association with cardiovascular disease and its major risk factors. Recognizing the limited number of RIPLs detected, the findings presented herein should be interpreted as preliminary and hypothesis-generating, intended to inform future larger-scale, powered studies.

## 2. Materials and Methods

This was a prospective and observational study involving individuals who underwent macular OCT imaging between 5 July and 5 October 2022. The study was conducted in accordance with the ethical principles outlined in the Declaration of Helsinki and was approved by the Ethics Committee of the European University of Madrid (Approval Code: CIPI/22.220; Approval Date: 29 July 2022). All participants provided written informed consent prior to inclusion, and ethical standards were maintained throughout all phases of the study.

### 2.1. Clinical Procedure

All participants underwent a 3D macular optical coherence tomography scan using the 6 × 6 mm cube protocol on the Topcon Maestro 2 system. Examinations were performed by a trained optometrist under standardized clinical conditions.

The Topcon Maestro 2 is a spectral-domain OCT device that provides 3D macular imaging with an axial resolution of approximately 6 µm and a transverse resolution of 20 µm. The 6 × 6 mm cube scan protocol used in this study captures 512 A-scans per B-scan across 128 B-scans, enabling high-resolution evaluation of retinal microstructures. Participants were instructed to fixate on the internal target during acquisition, and scans were repeated in cases of poor alignment or motion artifacts. All scans were performed under standard clinical lighting conditions and without pupil dilation [21].

Participants were selected based on predefined inclusion and exclusion criteria related to age, imaging quality, and ocular health status. A summary of these criteria is provided in Table 1.

OCT images were assessed using the integrated software of the Topcon Maestro 2 system (Topcon Corporation, Tokyo, Japan; Maestro2 software, version 2.20). Each scan was evaluated line-by-line, with the generation of retinal thickness maps and three-dimensional reconstructions. All images were independently assessed by two ophthalmologists and one optometrist under masked conditions regarding the patients’ clinical information. The evaluations were performed separately, without any communication between the reviewers, to ensure objectivity and minimize interpretation bias. The presence of a RIPL was confirmed only when full consensus was reached among all three evaluators. In cases where only two out of three graders agreed, the case was discussed collectively, and a final decision was made by full consensus. Prior to the image assessment phase, all evaluators participated in a brief calibration session using a reference set of OCT scans with and without RIPLs. This training aimed to standardize the application of diagnostic criteria and reduce subjectivity in the identification of subtle lesions. Although formal inter-rater agreement was not quantified statistically, preliminary concordance among the three graders was high, and the final RIPL determinations were based exclusively on full consensus.

Retinal ischemic perivascular lesions were identified according to previously published diagnostic criteria [22], defined by focal thinning of the inner nuclear layer and upward expansion of the outer nuclear layer in a perivascular area. The presence of RIPLs was only confirmed when all three observers reached a consensus.

**Figure 1:**
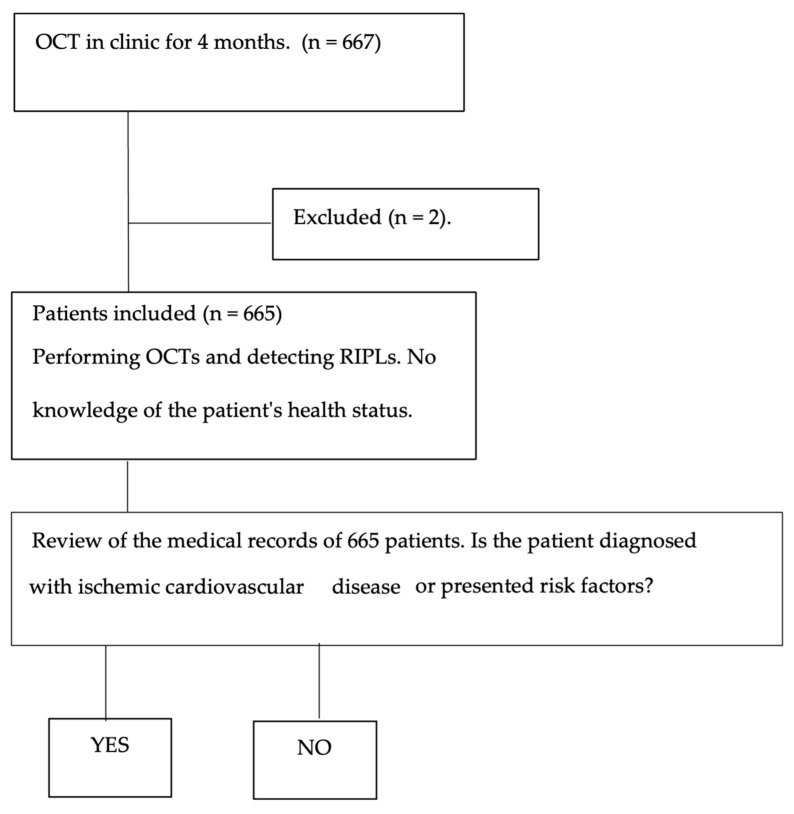
Flow chart of the method used in the identification of healthy and sick patients.

Once the image analysis was complete, the health information provided by the participants during the screening interview was reviewed. Based on this information, the population was divided into two groups: a “sick” group, including patients with diagnosed ischemic cardiovascular disease or risk factors (e.g., hypertension, dyslipidemia, coagulation disorders), and a “healthy” group, with no reported cardiovascular conditions. This classification process is summarized in Figure 1.

**Figure 2:**
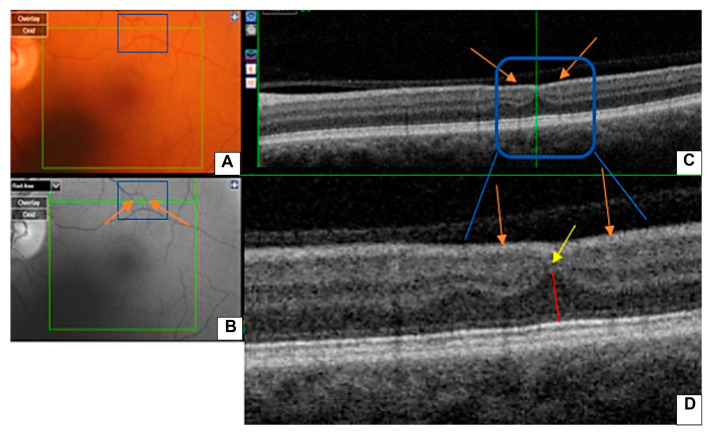
(**A**) Color retinography and (**B**) “red free” retinography. (**C**) B-scan and (**D**) enlarged b-scan, where the RIPL (blue frame) is observed. There is a focal contraction of the inner nuclear layer (yellow arrow) accompanied by an upward expansion of the darker outer nuclear layer (red line) in the area between two vessels (orange arrows).

Figure 2 illustrates a representative case of a RIPL, highlighting its characteristic anatomical features across different imaging modalities.

### 2.2. Statistical Analysis

All statistical analyses were performed using SPSS version 27. Categorical variables were analyzed using the Pearson Chi-square test, and associations between clinical variables and the presence of retinal ischemic perivascular lesions were assessed using logistic regression models. Odds ratios and 95% confidence intervals were calculated to estimate the strength of the association between RIPLs and selected cardiovascular risk factors. A significance level of *p* < 0.05 was considered statistically significant.

## 3. Results

Data from 665 participants were analyzed, with ages ranging from 40 to 90 years (interquartile range: 40–90) and a mean age of 60 ± 18 years. Of the total sample, 68.5% were female, and 31.5% were male. A total of 297 participants had ischemic cardiovascular disease or associated risk factors (diseased group), while 368 were classified as healthy based on the absence of reported cardiovascular conditions.

The overall prevalence of RIPLs was 0.75% (5 cases). Among the five positive cases, two patients showed unilateral lesions, and three presented bilateral involvement, which may reflect a more widespread vascular compromise. All RIPL cases were detected in the diseased group, representing a prevalence of 1.68% within that group and 0% in the healthy group. No RIPLs were observed in participants without cardiovascular risk factors. These findings are summarized in Table 2, which includes demographic data and RIPL prevalence by subgroup.

### Association Between RIPL Demographic and Clinical Variables

When stratified by age group, RIPL prevalence was 0% in individuals under 50 years, 0.56% in those aged 50–59, 0.54% in 60–69, 2.24% in 70–79, and 0% in participants aged 80 or older. No statistically significant difference was found in mean age between individuals with and without RIPLs (*p* > 0.05).

By sex, RIPLs were observed in 1.44% of men and 0.44% of women. This difference was not statistically significant (*p* = 0.39). The odds ratio for male sex was 0.90 (95% CI: 0.64–1.18).

Within the diseased group (*n* = 297), the most frequently reported conditions were hypertension (76.0%), dyslipidemia (44.1%), and heart failure (28.3%). Less frequent conditions included thrombosis (21.5%) and myocardial infarction (1.0%).

The presence of RIPLs was significantly more frequent in the diseased group compared to the healthy group (*p* = 0.044) (Table 3). Further analysis revealed statistically significant associations between RIPLs and hypertension (*p* < 0.001), dyslipidemia (*p* < 0.001), ischemic heart disease (*p* < 0.001), and thrombosis (*p* < 0.001). No significant associations were found with RIPLs and sex (*p* = 0.51), myocardial infarction (*p* = 0.15), or RIPLs themselves as predictors (*p* = 0.08).

## 4. Discussion

Our study demonstrated the exclusive presence of retinal ischemic perivascular lesions (RIPLs) in individuals with cardiovascular disease or associated risk factors. However, the overall prevalence was low, with only 1.68% of the diseased group exhibiting RIPLs. These findings suggest that although RIPLs may reflect localized microvascular damage, their utility as sensitive biomarkers for early detection of systemic ischemia in broad populations may be limited. This observation is partially consistent with previous reports, which have demonstrated associations between RIPLs and systemic vascular conditions, such as atrial fibrillation, coronary artery disease, and myocardial infarction [10,11,14,22]. However, significant discrepancies exist between our results and prior studies. For instance, Bakhoum et al. [14] reported an RIPL prevalence of approximately 34%, even among control participants without overt cardiovascular disease, while Bousquet et al. [22] identified a similarly high prevalence among patients with myocardial infarction. In contrast, we observed only five cases of RIPLs among 665 participants (0.75%), including zero cases in healthy individuals.

Several factors may account for these discrepancies. First, important differences in population characteristics must be considered. Prior studies often recruited individuals with known, advanced cardiovascular pathology or selected high-risk patients [14,22]. In contrast, our cohort consisted of a general adult screening population with a wider distribution of cardiovascular risk profiles and disease severity. The lower prevalence of advanced systemic vascular disease in our cohort may have contributed to the lower detection rate of RIPLs.

Second, methodological differences likely played a critical role. Our study applied a highly stringent definition of RIPLs, requiring unequivocal focal thinning of the inner nuclear layer adjacent to a retinal vessel accompanied by an upward expansion of the outer nuclear layer [11]. Moreover, the detection of RIPLs necessitated full consensus among three independent masked graders. In contrast, previous studies may have used more permissive criteria or relied on single-grader evaluations, potentially leading to the inclusion of subtle, borderline, or non-specific retinal changes under the RIPL definition [14,22].

In addition, differences in imaging modalities could have influenced detection rates. While we used the Topcon Maestro 2 OCT system and standardized 6 × 6 mm macular cube scans, other studies may have employed devices with higher axial resolution and more extensive scan coverage (e.g., widefield OCT) or utilized advanced segmentation algorithms [23] potentially enhancing the visibility of subtle lesions.

These methodological and population-based differences underscore the importance of establishing standardized criteria for RIPL diagnosis and evaluation. Without such standardization, inter-study comparisons remain challenging, and the true prevalence and significance of RIPLs may be difficult to ascertain.

Importantly, although the absolute prevalence of RIPLs was low, their exclusive presence among individuals with CVD or risk factors supports their potential relevance as indicators of systemic microvascular compromise [10,11]. Statistically significant associations were found between RIPLs and key cardiovascular risk factors, such as hypertension and dyslipidemia, consistent with the hypothesis that RIPLs may represent chronic manifestations of systemic endothelial dysfunction and chronic ischemia [24,25].

However, it is also important to recognize that no significant association was found between RIPLs and prior acute myocardial infarction in our cohort. This may reflect the low number of myocardial infarction events within the sample, the timing of retinal imaging relative to the ischemic event, or simply the small number of overall RIPL-positive cases, limiting statistical power. Future studies should address these possibilities through prospective designs and larger sample sizes.

Our findings suggest that while RIPLs may not be suitable as general screening biomarkers for early detection of cardiovascular disease, they may still hold clinical relevance. In particular, RIPLs could serve as markers of more advanced microvascular compromise or as indicators of cumulative vascular injury over time. Longitudinal studies would be essential to determine whether the presence of RIPLs predicts future cardiovascular or cerebrovascular events independent of traditional risk factors.

Our study possesses several strengths. It represents one of the few investigations assessing RIPL prevalence in a general adult screening population rather than a highly selected cardiovascular cohort. It employed rigorous, masked evaluation procedures with stringent consensus criteria, enhancing diagnostic specificity and minimizing interobserver bias. Furthermore, standardized imaging protocols were applied across all participants.

Nonetheless, several limitations must be acknowledged. First, the very low number of participants with RIPLs substantially limited the statistical power of our association analyses and precluded meaningful multivariate modeling. Second, our strict diagnostic criteria, while enhancing specificity, may have underestimated the true prevalence of subtle or early-stage RIPLs. Third, the use of standard 6 × 6 mm macular scans restricted the analysis to the central retina; peripheral RIPLs, if present, would have been missed. Future studies utilizing widefield OCT imaging may provide a more comprehensive assessment. Fourth, differences in imaging technologies, grader training, and RIPL definitions between studies complicate the direct comparison of prevalence rates across different reports. In particular, our findings highlight the urgent need for consensus definitions and standardized grading protocols for RIPLs in order to facilitate reproducibility and comparability across studies. Fifth, the ethnic homogeneity of our cohort limits generalizability to broader populations with diverse genetic and cardiovascular risk backgrounds. Finally, the cross-sectional nature of the study prevents any causal inferences regarding the relationship between RIPLs and systemic vascular disease. Longitudinal studies are needed to assess whether RIPLs precede, accompany, or follow the development of cardiovascular pathology. Moreover, data on body mass index, obesity status, and smoking history were not systematically collected, limiting a more comprehensive evaluation of cardiovascular risk factors.

Despite these limitations, our findings offer important insights into the nature of RIPLs and their potential clinical implications. Although not suitable as a general screening tool based on current evidence, the detection of RIPLs could be incorporated into comprehensive ophthalmologic assessments, particularly for patients at high cardiovascular risk. Further research should explore whether RIPLs, alone or combined with other retinal biomarkers, enhance cardiovascular risk prediction models beyond traditional clinical factors. Additionally, mechanistic studies investigating the pathophysiology of RIPLs at the microvascular level may help elucidate their origins and systemic correlates. Emerging imaging modalities, such as OCT angiography, could facilitate a deeper understanding of microvascular alterations associated with RIPLs and their potential progression over time.

## 5. Conclusions

This exploratory study provides preliminary evidence suggesting that retinal ischemic perivascular lesions are present predominantly in individuals with cardiovascular disease or associated risk factors such as hypertension and dyslipidemia. These findings support the hypothesis that RIPLs could serve as potential subclinical biomarkers of systemic ischemia. The detection of RIPLs through non-invasive optical coherence tomography in a general screening setting highlights their possible value for the early identification of vascular alterations related to cardiovascular disease. However, given the small number of cases observed, these results should be interpreted with caution. The extremely low prevalence of RIPLs in this sample (0.75%) severely limits statistical robustness, and the absence of data on important cardiovascular risk factors—such as smoking status, body mass index, and family history—restricts the comprehensiveness of the analysis. Additionally, the regional and ethnic homogeneity of the cohort reduces the generalizability of our findings to more diverse populations. Further large-scale, prospective studies are warranted to validate the clinical relevance of RIPLs in cardiovascular risk assessment and to better define their role within ophthalmological screening protocols.

## Figures and Tables

**Table 1:** Inclusion and exclusion criteria.

Inclusion Criteria	Exclusion Criteria
Adults aged 40 years or older	Poor-quality OCT images (signal strength < 6 or artifacts)
Underwent macular OCT scan with Topcon Maestro 2	Media opacities affecting image quality
Provided written informed consent	Fixation instability or segmentation errors
	History of ocular disease affecting retinal structure (e.g., diabetic retinopathy, retinal vein occlusion)
	Previous ocular surgeries (except uncomplicated cataract surgery)
	Refusal to participate

**Table 2:** Demographic data of the study population and RIPL prevalence.

Variable	Total (*n* = 665)	Diseased Group (*n* = 297)	Healthy Group (*n* = 368)
Mean age (SD)	61.64 ± 11.63	57.53 ± 11.05	66.67 ± 10.29
Female (%)	68.57%	71.04%	65.32%
Male (%)	31.43%	28.96%	34.68%
RIPLs (%)	0.75% (five cases)	1.68% (five cases)	0% (zero cases)

**Table 3:** Odds ratio of ischemic cardiovascular disease.

Covariates	Odds Ratio (CI 95%)	*p*-Value
Sex	0.9 (0.64–1.18)	0.51
RIPLs	13.3 (0.73–239.53)	0.08
Hypertension	49.4 (28.58–82.48)	<0.001
Ischemic heart disease	26.8 (10.69–66.75)	<0.001
Dyslipidemia	44.3 (19.16–101.60)	<0.001
Thrombosis	32.2 (9.97–103.13)	<0.001
Myocardial infarction	8.8 (0.45–167.6)	0.15

## Data Availability

Dataset available on request from the authors.
